# *Rickettsia conorii* subsp. *israelensis* infection in a pediatric patient presenting skin rash and abdominal pain: a case report from Southeast Iran

**DOI:** 10.1186/s12879-024-09002-y

**Published:** 2024-01-22

**Authors:** Ali Hosseininasab, Safoura MoradKasani, Ehsan Mostafavi, Neda Baseri, Maryam Sadeghi, Saber Esmaeili

**Affiliations:** 1 Research Center of Tropical and Infectious Diseases, Afzalipour Hospital, Kerman University of Medical Sciences, Kerman, Iran; 2 National Reference Laboratory for Plague, Tularemia and Q Fever, Research Centre for Emerging and Reemerging Infectious Diseases, Pasteur Institute of Iran, Kabudar Ahang, Akanlu, Hamadan, Iran; 3 Department of Epidemiology and Biostatics, Research Centre for Emerging and Reemerging Infectious Diseases, Pasteur Institute of Iran, Tehran, Iran; 4 Department of Bacteriology, Faculty of Medical Sciences, Tarbiat Modares University, Iran

**Keywords:** *Rickettsia conorii*, Mediterranean spotted fever, Pediatric, Child, Iran

## Abstract

**Background:**

The healthcare system in Iran appears to overlook Mediterranean spotted fever (MSF) as an endemic disease, particularly in pediatric cases, indicating the need for greater attention and awareness.

**Case presentation:**

A six-year-old patient with fever, abdominal pain, headache, skin rashes, diarrhea, vomiting, and black eschar (tache noire) from southeast Iran was identified as a rickettsiosis caused by *Rickettsia conorii* subsp. *israelensis* through clinical and laboratory assessments, including IFA and real-time PCR. The patient was successfully treated with doxycycline.

**Conclusions:**

Symptoms like rash, edema, eschar, and abdominal pain may indicate the possibility of MSF during the assessment of acute febrile illness, IFA and real-time PCR are the primary diagnostic methods for this disease.

## Introduction

*Rickettsia conorii* is a vector-borne pathogen that causes Mediterranean spotted fever (MSF). It is transmitted to humans primarily through tick bites [1]. Based on global data, the incidence of MSF exhibits a seasonal pattern, with the highest number of cases reported during the summer. This disease can be found across a significant geographical range that includes southern Europe, Africa, and the Middle East [2]. Fever, skin rash, and the presence of a black eschar (tache noire) at the site of the tick bite are common clinical presentations of MSF [3]. MSF can manifest a wide range of symptoms across various organs throughout the body, including atrial meningoencephalitis, sensorineural hearing loss, kidney failures, inflammation of the eyes, and other complications that affect multiple organs [4].

Based on recent studies, it appears that MSF is an endemic disease in Iran [5–7]. In a study, the MSF infection was found in 14.6% of Crimean-Congo Hemorrhagic Fever (CCHF) negative cases which all of them were missed by the healthcare system [8]. In the southeast region of Iran, it was found that 5% of the rural population had antibodies against *R. conorii* [9]. The epidemiology of MSF in Iran is unclear, and a significant number of clinical cases go undetected, indicating that it is a neglected disease. We present a case involving a child who was diagnosed with *R. conorii* subsp. israelensis infection. The diagnosis was made based on clinical and laboratory findings, and fortunately, the child responded well to the treatment.

## Case presentation

**Fig. 1:**
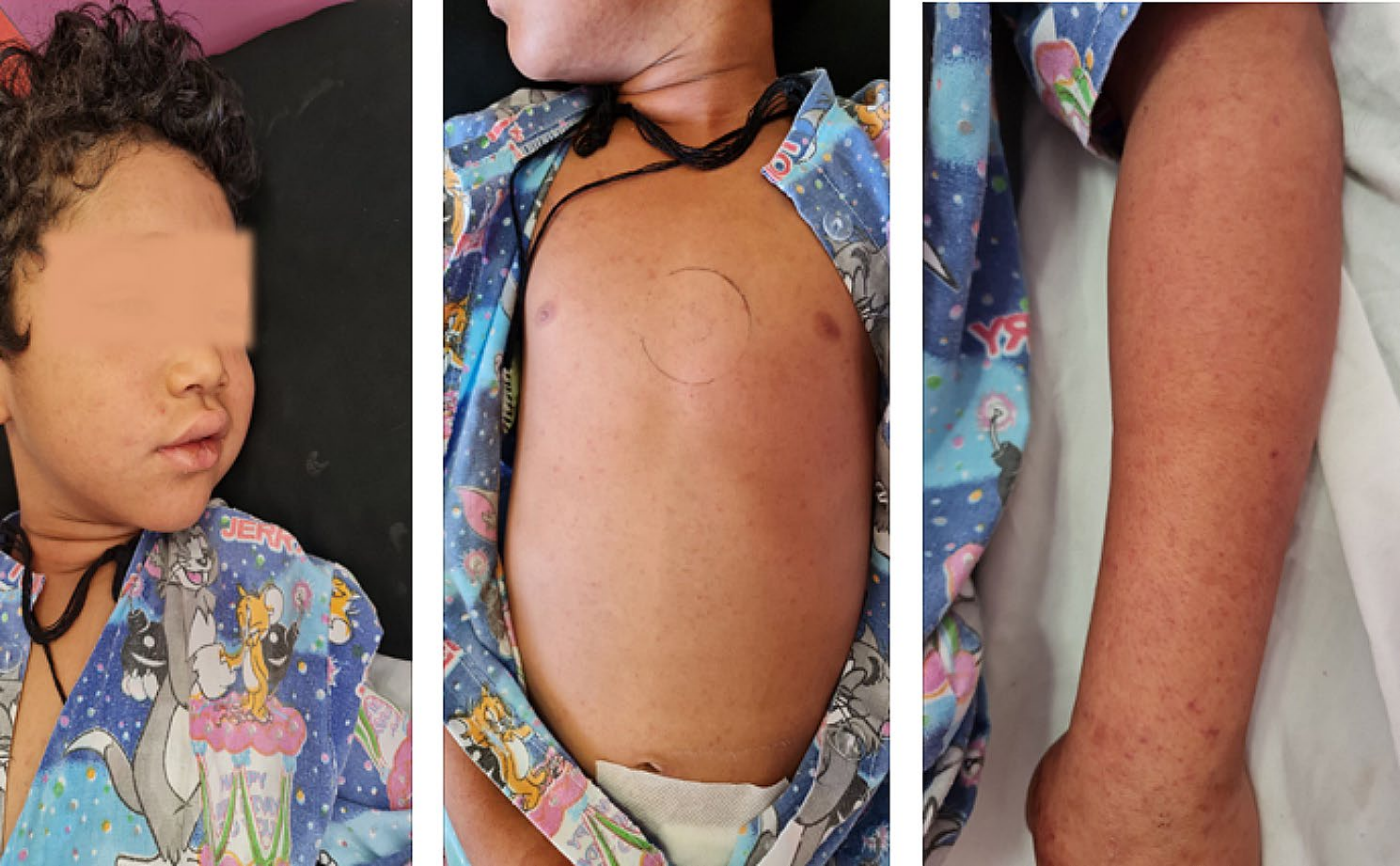
skin rashes of MSF in a six-year-old patient from southeast Iran, 2023

**Fig. 2:**
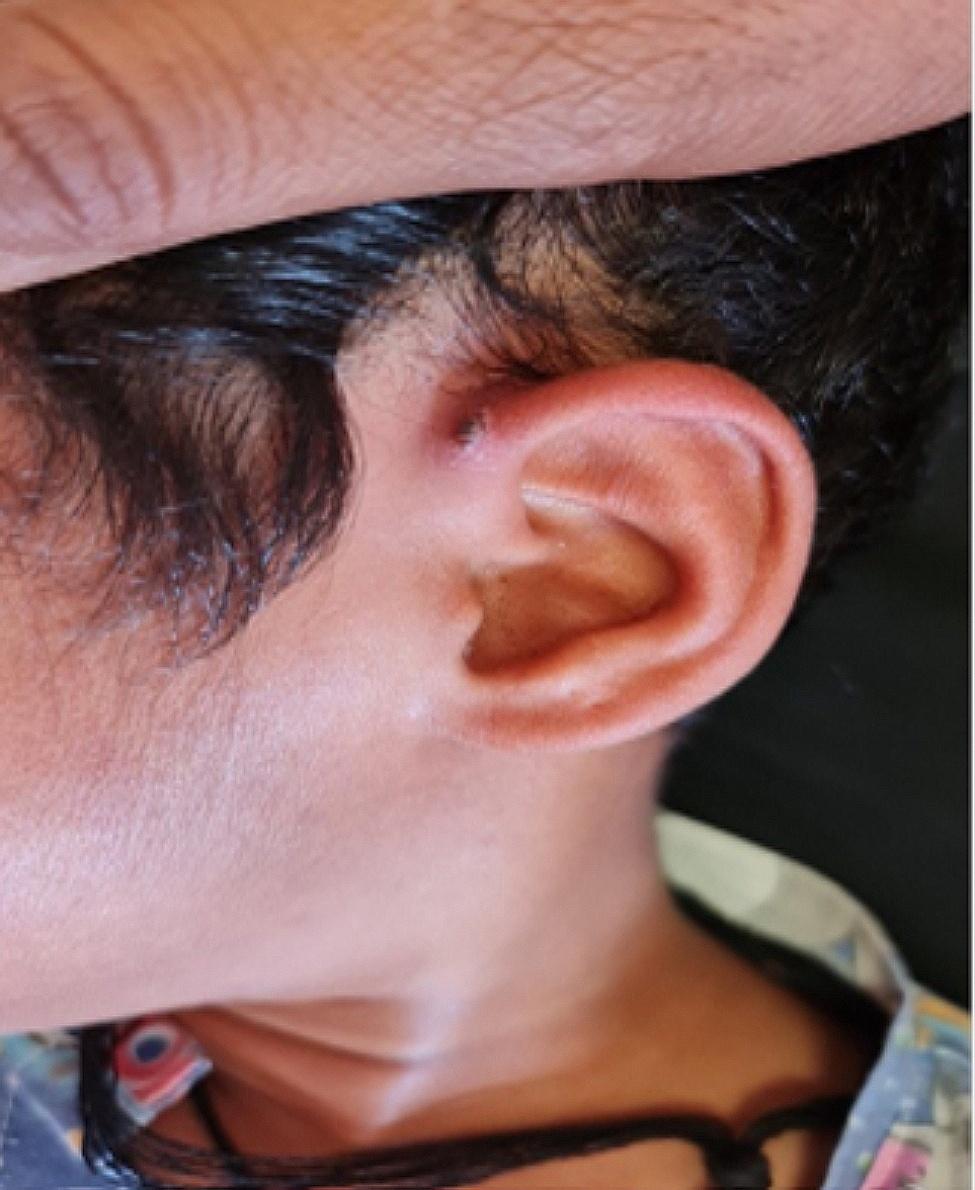
Black eschar with erythematous halo in front of the left ear of a six-year-old MSF patient from southeast Iran, 2023

A six-year-old boy residing in the Mohammadan City of Bampur County in Sistan and Baluchestan province (southeast Iran) was admitted to Afzalipour Hospital in Kerman City (a Referral Center for Infectious Diseases in Southeast Iran) on June 24, 2023. He had been suffering from fever, abdominal pain, and headache for seven days, and also developed skin rashes (Fig. 1). This patient had a history of tick bites one week before hospitalization. In addition, during the hospitalization examination, a black eschar with a surrounding erythematous halo measuring 1 cm in diameter was observed in front of the child’s left ear at the site of the tick bite (Fig. 2). Following the hospitalization, the patient gradually developed symptoms of diarrhea and vomiting. Physical examination uncovered non-pitting edema in the face and extremities, as well as tenderness in the right lower quadrant of the abdomen. Sonography examination pointed towards appendicitis; necessitating an appendectomy. Subsequent microscopic pathological investigation of the appendix tissue exhibited mucosal follicular hyperplasia, congestion, and infiltration of mononuclear cells. While admitted to the hospital, the patient experienced agitation, immobility, and reduced strength in the right upper limb. The results of a noninvasive Magnetic Resonance Imaging (MRI) were indicative of the expected findings.

Initial blood tests revealed mild anemia and thrombocytopenia. Hemoglobin (Hb), mean corpuscular volume (MCV) and mean corpuscular hemoglobin (MCH) levels were below the normal range as indicated in Table 1. Furthermore, elevated levels of the liver enzyme alanine aminotransferase (ALT) were detected beyond the standard range. On the second day of hospitalization, the serological results for brucellosis and salmonellosis were negative.

**Table 1:** Laboratory findings of MSF case during hospitalization in a six-year-old patient from southeast Iran, 2023

Days (hospitalization) /blood analysis	1	4	8
White blood cell (× 109/L)	Normal	12.1^*^	Normal
Hemoglobin (g/dl)	11.1^¥^	8.8^¥^	Normal
mean corpuscular hemoglobin (pg/cell)	25.6^¥^	25.4^¥^	Normal
mean corpuscular volume (fl.)	78.3^¥^	80.1^*^	Normal
Alanine aminotransferase (U/L)	51^*^	65^*^	Normal
Lymphocyte	16.3^¥^	Normal	Normal
Neutrophil	81.5*	Normal	Normal
Erythrocyte Sedimentation Rate (mm/hour)	18^*^	Normal	Normal
C-Reactive Protein (mg/dL)	117^*^	Normal	189*
Anisocytosis	Normal	Normal	mild
Urea (24)	Normal	Normal	165*

^*^increased, ^¥^decreased

On the fourth day of hospitalization, the patient underwent hematological tests again. The results indicated a continued decrease in Hb levels, with MCV and MCH values remaining below the normal range. Additionally, there was an observed increase in white blood cell (WBC) count. The ALT level tended to rise compared to the initial test conducted on the first day of hospitalization.

On the fifth day of hospitalization, the levels of C-reactive protein (CRP) and Erythrocyte Sedimentation Rate (ESR) were significantly elevated, surpassing the normal range. Particularly, the CRP level was more than 11 times higher than usual. On the sixth day of hospitalization, urine analysis was normal, and the urine culture was negative after 24 h.

On the eighth day of hospitalization, a 24-hour urine volume test was performed to determine the underlying cause of edema, revealing 165 mg of protein and 320 mg of creatinine in 1000 cc of urine. Subsequently, there was an observed increase in CRP levels, indicating ongoing inflammation. Additionally, hematological tests revealed mild anisocytosis in red blood cells (RBCs).

**Fig. 3:**
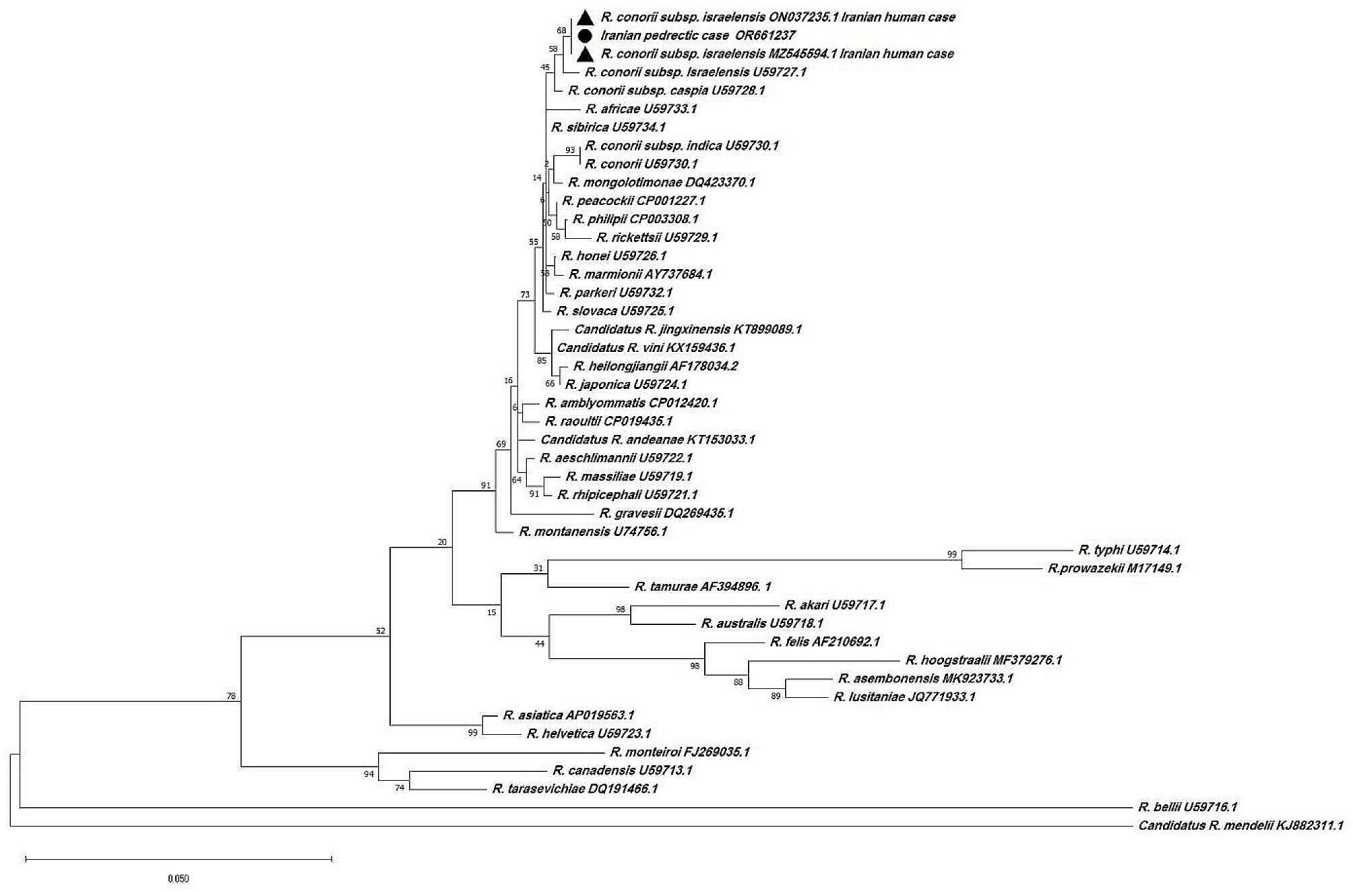
Phylogenetic analysis based on *Rickettsia* gltA gene sequencing and Maximum Likelihood method algorithm (Kimura 2-parameter model). The tests were performed with bootstrap (1000 repetitions) by MEGA X10.1 software. The current pediatric case (GenBank accession number: OR661237) and previous *Rickettsia conorii* subsp. *israelensis* human cases from Iran showed by • and $\blacktriangle$, respectively

On July 2nd, the patient’s serum and tissue samples, including a biopsy of a skin rash, were sent to the Research Centre for Emerging and Reemerging Infectious Diseases at the Pasteur Institute of Iran for further analysis and diagnosis. The anti-*R. conorii* IgM titer was determined to be 1:1536 through Indirect immunofluorescence (IFA) assay, indicating a high level of specific antibodies against the pathogen. Quantitative real-time polymerase chain reaction PCR (qPCR) tests targeting the 16 S rRNA gene of the serum samples yielded negative results for *Rickettsia* spp., suggesting the absence of the pathogen in the bloodstream. However, contrasting findings were observed in the qPCR analysis of the skin biopsy sample, which showed positive results for *Rickettsia* spp. (Table 2). By complementary phylogenetic studies that involved the amplification and sequencing of a specific gene (*glt*A) of *Rickettsia* spp., the infection was ultimately confirmed to be caused by *R. conorii* subsp. *israelensis* (Fig. 3).

**Table 2:** Primer sequences and product size used for detection and identification of *Rickettsia* spp

Gene target	Primer/probe name	Sequence (5’ to 3’)	Amplicon size (bp)	Ref
16 S rRNA	Rsp-Forward	CGC AAC CCT YAT TCT TAT TTGC	149	[10]
	Rsp-Reverse	CCT CTG TAA ACA CCA TTG TAGCA		
	Rsp-probe	6- FAM-TAA GAA AAC TGC CGG TGATAA GCCGGAG–TAMRA		
*glt*A	*glt*A-Forward	GCT CTT CTC ATC CTA TGG CTA TTA T	834	[10]
	*glt*A -Reverse	CAG GGT CTT CRT GCA TTT CTT		

On July 4, 2023, the patient received doxycycline (4 mg/kg/day) for five days, leading to a significant improvement in his condition. On 9th July of the same year, all symptoms completely disappeared, and the patient was discharged from the hospital. Two weeks later, the patient reported feeling completely fine and experiencing no ongoing issues.

## Discussion

*R. Conorii*, first identified by Conor in Tunisia in 1910, is associated with a disease known as MSF. This disease has multiple names depending on the region, such as Boutonneuse fever, Kenya Tick-Bite Fever, African Tick Typhus, Indian Tick Typhus, Israeli Spotted fever, and Marseilles fever [3].

In this report, we present a notable case of pediatric rickettsiosis caused by *R. conorii* subsp. *israelensis* in southeastern Iran. It is worth emphasizing that documented cases of rickettsial infection have primarily been observed in Southeast Iran [5–7]. It appears that MSF continues to be an endemic disease in this region, yet it remains a neglected infectious disease [8, 9].

Despite tick bites often going unnoticed, our patient presented with a Tache noire, along with symptoms such as fever, headache, rashes, agitation, immobility, weakened limbs, abdominal pain, and gastrointestinal manifestations.

Common symptoms of this disease include headaches, malaise, and fever. The characteristic rash usually emerges between the third and fifth days of fever, starting with lesions on the extremities and gradually spreading to the trunk, neck, face, buttocks, and palms within 24–36 h. In rare cases, *R. conorii* infection can severely affect the central nervous system in adults, but it is less common in children [3].

Abdominal and hepatic involvements are commonly observed in many cases of MSF. This can sometimes lead to confusion when diagnosing other illnesses. It is crucial to note that abdominal pain could be a symptom of MSF and should be carefully taken into consideration [11].

Diagnosing MSF can be challenging due to its non-specific clinical symptoms. Late diagnosis can result in various complications, including neurological impairment, liver damage, respiratory failure, multi-organ failure, and even death [4]. Routine laboratory procedures such as blood cultures are unable to isolate *R. conorii*. Diagnosis relies on clinical features, geographic background, and epidemiological considerations [12]. In this case, IFA, the gold standard diagnostic test, was used to detect IgM antibodies specific to the MSF infection. The patient’s laboratory results indicated thrombocytopenia, elevated liver enzyme levels, and hemoglobin levels, which are common abnormalities observed in MSF patients [14]. In the early stages of MSF, serological tests are not reliable for diagnosis because of the delayed development of antibodies against *R. conorii*. At least two serum samples collected two–four weeks apart during the acute and convalescent phases of illness are required for definitive diagnosis. Seroconversion or a fourfold or greater increase in antibody titer between acute and convalescent samples confirms acute or recent infection [4]. Molecular tests are currently widely used for detecting rickettsiosis due to their high sensitivity and specificity compared to serological tests [15]. For definitive diagnosis, a skin biopsy sample was obtained and used for molecular testing, which is considered the most effective option [11]. In current our case, the molecular test was negative on the blood sample, but the results of qPCR on a skin biopsy were positive. This case illustrates the greater value of a sample of infected tissue than peripheral blood for PCR diagnosis. It should be pointed out that a swab taken from the base of an eschar is highly sensitive and less invasive than a skin biopsy [16].

To prevent severe complications and fatal outcomes associated with tick-borne bacterial disease therapy, definitive diagnostic test results should not be delayed. The patient received doxycycline before receiving definitive diagnostic test results from the Research Center for Emerging and Reemerging Infectious Diseases at the Pasteur Institute of Iran, and his condition improved. Doxycycline is the preferred medication for bacterial tick-borne diseases and is commonly recommended as the primary treatment for MSF to prevent fatal outcomes [2].

## Conclusion

The focus of this article is to emphasize the importance of raising awareness about MSF in southeastern Iran. While symptoms like rash, edema, eschar, and abdominal pain may indicate the possibility of MSF during the assessment of acute febrile illness, IFA and real-time PCR are the primary diagnostic methods for this disease.

## Data Availability

All data generated or analyzed in this study are included in this published article.

## Abbreviations

MSF: Mediterranean spotted fever
CCHF: Crimean-Congo hemorrhagic fever
IFA: Indirect immunofluorescence
MRI: Noninvasive Magnetic Resonance Imaging
RBCs: Red blood cells
qPCR: Quantitative real-time polymerase chain reaction PCR
MCV: Mean corpuscular volume
MCH: Mean corpuscular hemoglobin
ALT: Alanine aminotransferase
ESR: Sedimentation Rate
